# Resistance training and total and site-specific cancer risk: a prospective cohort study of 33,787 US men

**DOI:** 10.1038/s41416-020-0921-8

**Published:** 2020-06-04

**Authors:** Leandro F. M. Rezende, Dong Hoon Lee, NaNa Keum, Kana Wu, José Eluf-Neto, Fred K. Tabung, Edward L. Giovannucci

**Affiliations:** 1 Universidade Federal de São Paulo, Escola Paulista de Medicina, Departamento de Medicina Preventiva, Sao Paulo, Brazil; 2 Departamento de Medicina Preventiva, Faculdade de Medicina FMUSP, Universidade de Sao Paulo, Sao Paulo, Brazil; 3 Department of Nutrition, Harvard T.H. Chan School of Public Health, Boston, MA USA; 4 Department of Food Science and Biotechnology, Dongguk University, Goyang, South Korea; 5 Division of Medical Oncology, Department of Internal Medicine, The Ohio State University College of Medicine, Columbus, OH USA; 6 Department of Epidemiology, Harvard T.H. Chan School of Public Health, Boston, MA USA; 7 Channing Division of Network Medicine, Department of Medicine, Brigham and Women’s Hospital and Harvard Medical School, Boston, MA USA

**Keywords:** Epidemiology, Cancer epidemiology

## Abstract

**Background:**

Muscle-strengthening activities have been recommended for health benefits. However, it is unclear whether resistance training is associated with cancer risk, independent of total physical activity.

**Methods:**

A prospective cohort study followed 33,787 men from the Health Professionals Follow-up Study (1992–2014). Cumulative average of resistance training (hours/week) was assessed through biennial questionnaires up to 2 years before cancer diagnosis. Cox regression model was used to estimate the hazard ratio (HR) and 95% confidence intervals (CI).

**Results:**

During 521,221 person-years of follow-up, we documented 5,158 cancer cases. Resistance training was not associated with total cancer risk (HR per 1-h/week increase: 1.01; 95% CI 0.97, 1.05). We found an inverse association between resistance training and bladder cancer (HR per 1-h/week increase: 0.80; 95% CI 0.66, 0.96) and kidney cancer (HR per 1-h/week increase 0.77; 95% CI 0.58, 1.03; P_trend_ = 0.06), but the association was marginal for the latter after adjustment for confounders and total physical activity. Compared to participants engaging in aerobic activities only, combined resistance training and aerobic activities showed stronger inverse associations with kidney cancer risk.

**Conclusions:**

Resistance training was associated with lower risk of bladder and kidney cancers. Future studies are warranted to confirm our findings.

## Background

Moderate to vigorous intensity, aerobic physical activity (MVPA) has been linked with lower risk of several types of cancer, including breast, colon and endometrial;^1,2^ and, more recently, kidney, bladder, oesophageal and stomach cancers.^3,4^ The 2018 Physical Activity Guidelines for Americans recommended 150–300 min/week of moderate intensity or 75–150 min/week of vigorous intensity activity to reduce risk of cancer,^5^ although the optimum amount (dose) and types of physical activity are unknown.^4,6^

Muscle-strengthening activities, such as weight lifting or resistance training, at least 2 days/week has also been recommended for health benefits, such as reducing blood pressure and maintaining lean body mass.^5^ However, there is limited epidemiological evidence supporting the effect of resistance training on cancer risk. In addition, it is unclear if, for the same amount of total physical activity, combined resistance training and aerobic activities provide additional cancer risk reduction compared to engaging in aerobic activities only.^4^ To our knowledge, only one large prospective study investigated the association between muscle-strengthening activities and cancer risk.^7^ In that study, weight training was associated with a lower risk of colon, and a trend towards lower risk of kidney cancer after adjustment for MVPA.^7^ However, weight lifting was measured only once over time, which could lead to measurement error of the exposure and thus regression dilution bias.^7,8^

In the current study, we examined the association of cumulative average of resistance training over time with total and site-specific cancer risk in the Health Professionals Follow-up Study (HPFS). We also determined whether these associations varied by potential effect modifiers (attained age, body mass index (BMI), and smoking), and considered the joint association of resistance training and total physical activity with risk of site-specific cancers.

## Methods

### Study population

The HPFS is an ongoing prospective cohort study that enrolled 51,529 US male health professionals in 1986.^9^ At the enrolment, participants aged 40–75 years completed a questionnaire on demographic, lifestyle and health-related factors. Biennial follow-up questionnaires have been administered since then, reaching over 90% response rate. The study protocol was approved by the institutional review board of the Harvard T.H. Chan School of Public Health and those of participating cancer registries as required.

In this study, the baseline was defined as 1990 when participation in weight training or resistance exercises was first assessed. At baseline (*n* = 47,678), we excluded men having prior cancer diagnosis (*n* = 3001) or missing data on resistance training (*n* = 10,890). The final analytical cohort included 33,787 men.

### Assessment of resistance training

In 1990 and every 2 years thereafter, participants reported average time per week (zero, 1–4 min, 5–19 min, 20–59 min, 1 h, 1–1.5 h, 2–3 h, 4–6 h, 7–10 h, ≥11 h) spent in the following recreational activities: walking or hiking outdoors, jogging, running, bicycling, lap swimming, tennis, squash or racquetball, calisthenics or rowing, weight lifting or nautilus or weight machine and heavy outdoor work.

Resistance training was defined by average hours a week in weight lifting or nautilus or weight machine. We also assigned metabolic equivalent of task (MET) for all other recreational activities and heavy outdoor work to classify intensity, and then summed up to obtain total physical activity, except resistance training, in MET-h/week.^10^

### Assessment of covariates

Biennial questionnaires included information on potential confouders: age, race, height, BMI, family history of cancer, physical exam in past 2 years, history of colonoscopy or sigmoidoscopy, prostate-specific antigen (PSA) test in past two years, smoking, regular aspirin use, and multivitamin use, total energy intake, alcohol consumption, red and processed meat intake and Alternate Healthy Eating Index.^11^ Dietary information was obtained through a validated semiquantitative food frequency questionnaire every 4 years.^12^

### Ascertainment of cancer cases

Participants (or the next of kin for those who died) were asked whether they were diagnosed with cancer in the previous two years through biennial follow-up questionnaires up to 2014. Self-reported cancer diagnosis was confirmed by physicians, who reviewed medical records blinded to the participants’ exposure status. Importantly, about 90% of the medical records were obtained upon request. For nonrespondents, we searched the National Death Index to identify those who may have died from cancer. Over 98% of deaths were ascertained from the follow-up. Cancer types were confirmed using the International Classification of Disease 9 (ICD-9). We included only cancer sites that had at least 100 cases diagnosed in the period: colon (ICD-9 153), advanced prostate cancer (ICD-9 185; i.e. advanced prostate cancer was defined as stage T3b, T4, N1, M1 or death from prostate cancer), lung (ICD-9 162), bladder (ICD-9 188), lymphoma (ICD-9 200, 202, 204), pancreas (ICD-9 157), kidney (ICD-9 189), leukaemia (ICD-9 205–207), multiple myeloma (ICD-9 203) and oesophagus (ICD-9 150). We included advanced prostate cancer only because nonadvanced incident cancers are relatively benign and prone to detection bias.^13^ In addition, body fatness, a potential mediator for the association between resistance training and prostate cancer, has been associated with advanced prostate cancer only.^14^

### Statistical analysis

Hazard ratios (HR) and 95% confidence intervals (CI) were estimated through Cox proportional hazard models using age as time scale with stratification by calendar time. Person-time of follow-up was calculated starting from the return date of the baseline questionnaire until the cancer diagnosis, death or end of the study (January 2014), whichever came first. For resistance training, total physical activity, and covariates, we calculated their cumulative average using all available biennial follow-up questionnaires. We calculated cumulative average variables as the mean of all available information from baseline through each new follow-up questionnaire, to better reflect participants long-term exposure and reduce measurement error. In addition, we added a 2-year latency period separating the exposure and follow-up time at risk to reduce potential bias due to reverse causation. For instance, the average hours of resistance training in 1990 was used for follow-up from 1992 to 1994. Similarly, the average hours of resistance training in 1990 and 1992 (cumulative average) was used for follow-up from 1994 to 1996. Multivariable models were built based on prior knowledge of deleterious or beneficial factors for cancer risk.^1,7,9^ The first multivariable model used age (month) as time scale with stratification by calendar time, and adjusted for race (white or non-white), height (continuous), family history of cancer (yes or no), physical exam in past 2 years (yes or no), history of colonoscopy or sigmoidoscopy (yes or no), smoking in pack years (never smoker, 1–4.9, 5–19.9, 20–39.9 or ≥40), regular aspirin use (yes or no), multivitamin use (yes or no), alcohol consumption (0, 0.1–4.9, 5.0–14.9, 15.0–29.9 or ≥30 g/d), red and processed meat intake (quintiles), Alternate Healthy Eating Index (quintiles) and PSA test in past 2 years (yes or no). Model 2 additionally adjusted for total physical activity, except resistance training (quintiles). Model 3 additionally adjusted for total energy intake (quintiles) and body mass index (quintiles), which are potential mediators of the association between resistance training and cancer risk. We considered for main findings, HR and 95% CI derived from multivariable model 2, which provides information on the association between resistance training and cancer risk independent of confounders and total physical activity (excluding resistance training).

Resistance training was analysed as both continuous (per hour a week) and categorical (none, <1 h/week, ≥1 h/week) based on the distribution of exposure. We also performed sensitivity analysis using different cut-offs for resistance training (none vs. any). Total physical activity was categorised based on its distribution by comparing lowest tertile (<16 MET-h/week) vs. top-two tertiles (≥16 MET-h/week).

For the cancer sites presenting statistically significant associations in one of the multivariable models, we performed subgroup analyses to examine the association between resistance training and cancer risk by attained age, smoking and BMI. These variables have been suggested to be potential effect modifiers for the association between physical activity/resistance training and cancer risk.^2,3,7^ To formally test interactions, we included the multiplicative term (cross-product term) of resistance training (both binary and continuous) and each effect modifier into the model and used Wald test to assess statistical significance. We also evaluated whether, for the same amount of total physical activity, combined resistance training and aerobic activity provide additional cancer risk reduction compared to engaging in aerobic activities only. Thus, we performed joint association of resistance training (none vs. any) and total physical activity (high vs. low MET-h/week) with risk of site-specific cancers. In the joint analysis, none resistance training and low total physical activity was defined as reference group.

All analyses were performed using SAS statistical software (version 9.4; SAS Institute Inc).

## Results

Over 521,221 person-years of follow-up, we ascertained 5158 cancer cases. For site-specific cancer cases we ascertained: colon, 700; advanced prostate, 657; lung, 595; bladder, 505; lymphoma, 484; pancreas, 233; kidney, 212; leukaemia, 188; multiple myeloma, 112; oesophagus, 103. Compared to participants reporting no resistance training, those engaging in ≥1 h/week were younger and more likely having family history of cancer, physical examination and PSA test in the past 2 years; being current users of multivitamin, physically active, never smokers, leaner and to eat healthier diets, based on AHEI scores (Table 1).

**Table 1:** Age-standardised characteristics of person-years according to resistance training in the Health Professionals Follow-up Study (1992–2014).

Characteristic	Resistance training		
	None	<1 h/week	1+ h/week
Resistance training (h/week), median (IQR)^a^	0	0.3 (0.1–0.5)	1.6 (1.3–2.5)
Participants at baseline	28,224	1932	3631
Person-years	336,057	125,143	60,021
Age (years)	66.7 (10.3)	66.9 (9.6)	63.6 (9.5)
White (%)	90.9	90.8	90.3
Family history of cancer (%)	32.4	36.9	34.8
Height (cm)	178.2 (6.7)	178.7 (6.6)	179.0 (6.6)
Body mass index	26.0 (3.4)	25.7 (3.1)	25.2 (2.9)
Overweight (%)	47.3	45.5	41.8
Obese (%)	10.5	8.1	6.1
Physical examination in past 2 years (%)	70.2	77.2	74.2
History of colonoscopy or sigmoidoscopy (%)	46.2	64.1	59.9
Prostate-specific antigen test in past 2 years (%)	46.7	62.3	57.5
Regular aspirin use (%)	46.9	49.0	46.1
Current use of multivitamin (%)	48.2	61.3	61.6
Total physical activity (MET-h/week)^b^	25.6 (22.1)	34.1 (23.4)	51.2 (31.1)
Never smoker (%)	51.9	55.0	53.8
No of pack years among ever smokers	25.0 (20.0)	18.8 (16.9)	18.4 (16.1)
Calorie intake (kcal/day)	1978 (533)	1990 (519)	2003 (512)
Alcohol intake (g/day)	10.8 (13.7)	11.2 (12.5)	11.0 (11.7)
Red and processed meat (no of servings/week)	6.7 (4.3)	5.8 (3.8)	5.2 (3.7)
Alternate Healthy Eating Index	47.1 (9.6)	49.8 (9.2)	52.2 (9.8)

Values are means (SD) or percentages and are standardised to the age distribution of the study population (except for age). All Information was updated over the follow-up period.

*IQR* interquartile range, *MET* metabolic equivalent task.

^a^Ranges of resistance training (h/week): ‘None’ (0 h/week); <1 h/week’ (0.1–0.9 h/week); 1+ h/week (1–15.5 h/week) .

^b^Total physical activity including resistance training.

In the multivariable model 1, resistance training was not associated with risk of total cancer (Table 2). For site-specific cancer, we found an inverse association with risk of bladder cancer and kidney cancer. Participants reporting ≥1 h/week had a 36% lower risk of bladder cancer (HR 0.64; 95% CI 0.44, 0.93) and a 48% lower risk of kidney cancer (HR 0.52; 95% CI 0.29, 0.94) compared to none resistance training group. The HRs per hour increase of resistance training were 0.81 (95% CI 0.68, 0.97) for bladder cancer and 0.74 (95% CI 0.56, 0.99) for kidney cancer. After adding total physical activity, except resistance training, into the model (multivariable model 2), the magnitude of the associations was slightly attenuated and kidney cancer was marginally associated (HR 0.57; 95% CI 0.31, 1.03; P for trend = 0.06). For bladder cancer, the HR comparing ≥1 h/week vs. none resistance training was 0.61 (95% CI 0.42, 0.89). Finally, adding total energy intake and BMI into the model had minimal influence for bladder and kidney cancer results, but for colon cancer, a positive association became marginally apparent (HR 1.32; 95% CI 1.01, 1.72; P for trend = 0.05). Resistance training was not associated with the other eight types of cancers.

**Table 2:** Resistance training and risk of total and site-specific cancer in the Health Professionals Follow-up Study (1992–2014).

HR (95% CI)	Resistance training			P-trend^b^	Per 1-h/week increase
	None	<1 h/week	1+ h/week		
*Total cancer* (*n* = 5158)^a^					
Event	3530	1 154	474		
Person-years	336,057	125,143	60,021		
Multivariable 1	1 (ref)	0.97 (0.90, 1.04)	0.97 (0.88, 1.07)	0.53	1.00 (0.96, 1.04)
Multivariable 2	1 (ref)	0.97 (0.90, 1.05)	0.98 (0.89, 1.09)	0.76	1.01 (0.97, 1.05)
Multivariable 3	1 (ref)	0.98 (0.91, 1.05)	0.99 (0.90, 1.10)	0.88	1.01 (0.97, 1.05)
*Cancer sites*					
Colon cancer (*n* = 700)					
Event	496	133	71		
Multivariable 1	1 (ref)	0.92 (0.75, 1.13)	1.23 (0.95, 1.60)	0.14	1.10 (1.00, 1.20)
Multivariable 2	1 (ref)	0.94 (0.76, 1.15)	1.28 (0.98, 1.67)	0.07	1.11 (1.02, 1.22)
Multivariable 3	1 (ref)	0.94 (0.77, 1.16)	1.32 (1.01, 1.72)	0.05	1.12 (1.02, 1.22)
Advanced prostate cancer (*n* = 657)					
Event	487	116	54		
Multivariable 1	1 (ref)	0.95 (0.76, 1.19)	0.89 (0.66, 1.19)	0.43	0.95 (0.84, 1.07)
Multivariable 2	1 (ref)	0.95 (0.76, 1.19)	0.88 (0.66, 1.19)	0.41	0.95 (0.84, 1.07)
Multivariable 3	1 (ref)	0.96 (0.77, 1.20)	0.89 (0.66, 1.19)	0.43	0.95 (0.84, 1.07)
Lung cancer (*n* = 595)					
Event	447	109	39		
Multivariable 1	1 (ref)	0.85 (0.68, 1.07)	0.87 (0.62, 1.22)	0.36	0.91 (0.78, 1.07)
Multivariable 2	1 (ref)	0.87 (0.69, 1.09)	0.90 (0.64, 1.28)	0.51	0.93 (0.79, 1.09)
Multivariable 3	1 (ref)	0.86 (0.69, 1.09)	0.90 (0.63, 1.27)	0.48	0.93 (0.79, 1.09)
Bladder cancer (*n* = 505)					
Event	345	128	32		
Multivariable 1	1 (ref)	0.97 (0.78, 1.21)	0.64 (0.44, 0.93)	0.02	0.81 (0.68, 0.97)
Multivariable 2	1 (ref)	0.94 (0.75, 1.17)	0.61 (0.42, 0.89)	0.01	0.80 (0.66, 0.96)
Multivariable 3	1 (ref)	0.94 (0.75, 1.18)	0.61 (0.42, 0.90)	0.01	0.80 (0.66, 0.96)
Lymphoma (*n* = 484)					
Event	316	118	50		
Multivariable 1	1 (ref)	1.01 (0.80, 1.27)	1.06 (0.78, 1.45)	0.71	1.06 (0.94, 1.18)
Multivariable 2	1 (ref)	1.02 (0.81, 1.29)	1.08 (0.79, 1.49)	0.62	1.06 (0.95, 1.19)
Multivariable 3	1 (ref)	1.02 (0.81, 1.29)	1.08 (0.79, 1.50)	0.62	1.07 (0.95, 1.19)
Pancreatic cancer (*n* = 233)					
Event	153	57	23		
Multivariable 1	1 (ref)	1.06 (0.76, 1.47)	1.08 (0.68, 1.71)	0.75	0.98 (0.80, 1.19)
Multivariable 2	1 (ref)	1.11 (0.79, 1.55)	1.18 (0.74, 1.89)	0.49	1.01 (0.83, 1.23)
Multivariable 3	1 (ref)	1.13 (0.81, 1.57)	1.22 (0.76, 1.96)	0.40	1.01 (0.84, 1.23)
Kidney cancer (*n* = 212)					
Event	147	52	13		
Multivariable 1	1 (ref)	0.86 (0.61, 1.21)	0.52 (0.29, 0.94)	0.03	0.74 (0.56, 0.99)
Multivariable 2	1 (ref)	0.88 (0.62, 1.25)	0.57 (0.31, 1.03)	0.06	0.77 (0.58, 1.03)
Multivariable 3	1 (ref)	0.89 (0.63, 1.26)	0.58 (0.32, 1.04)	0.07	0.78 (0.58, 1.04)
Leukemia (*n* = 188)					
Event	129	41	18		
Multivariable 1	1 (ref)	0.78 (0.54, 1.14)	0.91 (0.54, 1.52)	0.70	1.05 (0.87, 1.27)
Multivariable 2	1 (ref)	0.80 (0.55, 1.18)	0.99 (0.58, 1.68)	0.95	1.09 (0.90, 1.32)
Multivariable 3	1 (ref)	0.81 (0.55, 1.19)	1.00 (0.59, 1.70)	0.99	1.09 (0.90, 1.32)
Multiple myeloma (*n* = 112)					
Event	75	27	10		
Multivariable 1	1 (ref)	0.98 (0.61, 1.58)	0.89 (0.45, 1.79)	0.75	0.84 (0.58, 1.21)
Multivariable 2	1 (ref)	0.98 (0.60, 1.59)	0.91 (0.45, 1.85)	0.80	0.85 (0.58, 1.23)
Multivariable 3	1 (ref)	0.99 (0.61, 1.60)	0.93 (0.46, 1.89)	0.84	0.85 (0.59, 1.24)
Oesophageal cancer (*n* = 103)					
Event	69	28	6		
Multivariable 1	1 (ref)	1.29 (0.79, 2.11)	0.70 (0.30, 1.68)	0.47	0.91 (0.64, 1.29)
Multivariable 2	1 (ref)	1.28 (0.78, 2.10)	0.72 (0.30, 1.74)	0.51	0.92 (0.65, 1.30)
Multivariable 3	1 (ref)	1.27 (0.77, 2.09)	0.71 (0.30, 1.72)	0.48	0.91 (0.64, 1.30)

Age-adjusted models: Cox regression models using age (month) as time scale with stratification by calendar time (year).

Multivariable 1 models: Additionally adjusted for race (white or non-white), height (continuous), family history of cancer (yes or no), physical exam in past two years (yes or no), history of colonoscopy or sigmoidoscopy (yes or no), smoking in pack years (never smoker, 1–4.9, 5–19.9, 20–39.9 or ≥40), regular aspirin use (yes or no), multivitamin use (yes or no), alcohol consumption (0, 0.1–4.9, 5.0–14.9, 15.0–29.9, or ≥30 g/d), red and processed meat intake (quintiles), Alternate Healthy Eating Index (quintiles) and prostate-specific antigen test in past 2 years (yes or no).

Multivariable 2 models: Additionally adjusted for total physical activity except for resistance training (quintiles).

Multivariable 3 models: Additionally adjusted for total energy intake (quintiles) and body mass index (quintiles).

^a^Included only aggressive prostate cancer as total cancer.

^b^P-trend was calculated using medians of resistance training categories as an ordinal variable in the models.

### Subgroup analysis

For the cancer sites associated with resistance training in one of the previous multivariable models (i.e. kidney, bladder and colon), we performed subgroup analyses to examine whether the associations varied by attained age, BMI and smoking (Table 3). We did not observe strong evidence of effect modifications. The inverse association between resistance training and kidney cancer was restricted to never smokers (any vs. none resistance training: HR 0.59; 95% CI 0.34, 0.99) and ≥65 years participants (any vs. none resistance training: HR 0.62; 95% CI 0.41, 0.94). For bladder cancer, inverse association was observed among ≥65 years participants (HR per hour: increase of resistance training 0.75; 95% CI 0.59, 0.95), overweight (HR per hour increase of resistance training: 0.69; 95% CI 0.51, 0.93) and ever smokers (HR per hour increase of resistance training: 0.74; 95% CI 0.57, 0.96).

**Table 3:** Subgroup analyses of resistance training and risk of site-specific cancer in the Health Professionals Follow-up Study (1992–2014).

HR (95% CI)	Resistance training		P-interaction^a^	Per 1-h/week increase	P-interaction^b^
	None	Any			
*Age*					
Colon cancer					
<65 years (*n* = 192)	1 (ref)	0.97 (0.70, 1.34)	0.54	1.13 (1.01, 1.28)	0.94
≥65 years (*n* = 508)	1 (ref)	1.06 (0.86, 1.32)		1.10 (0.96, 1.26)	
Bladder cancer					
<65 years (*n* = 101)	1 (ref)	0.95 (0.61, 1.48)	0.73	0.92 (0.69, 1.21)	0.36
≥65 years (*n* = 404)	1 (ref)	0.83 (0.65, 1.05)		0.75 (0.59, 0.95)	
Kidney cancer					
<65 years (*n* = 66)	1 (ref)	1.36 (0.77, 2.39)	0.15	1.02 (0.76, 1.36)	0.10
≥65 years (*n* = 146)	1 (ref)	0.62 (0.41, 0.94)		0.55 (0.33, 0.90)	
*BMI*					
Colon cancer					
<25 kg/m^2^ (*n* = 259)	1 (ref)	0.96 (0.71, 1.29)	0.21	1.08 (0.93, 1.25)	0.66
≥25 kg/m^2^ (*n* = 441)	1 (ref)	1.15 (0.91, 1.45)		1.13 (1.01, 1.27)	
Bladder cancer					
<25 kg/m^2^ (*n* = 222)	1 (ref)	0.79 (0.57, 1.08)	0.41	0.86 (0.68, 1.10)	0.31
≥25 kg/m^2^ (*n* = 283)	1 (ref)	0.89 (0.67, 1.18)		0.69 (0.51, 0.93)	
Kidney cancer					
<25 kg/m^2^ (*n* = 76)	1 (ref)	0.70 (0.39, 1.27)	0.61	0.75 (0.44, 1.26)	0.44
≥25 kg/m^2^ (*n* = 136)	1 (ref)	0.85 (0.57, 1.28)		0.77 (0.54, 1.09)	
*Smoking status*					
Colon cancer					
Never smoker (*n* = 338)	1 (ref)	0.96 (0.74, 1.25)	0.46	1.04 (0.89, 1.22)	0.13
Ever smoker (*n* = 362)	1 (ref)	1.11 (0.86, 1.43)		1.14 (1.03, 1.27)	
Bladder cancer					
Never smoker (*n* = 187)	1 (ref)	0.94 (0.67, 1.31)	0.11	0.83 (0.63, 1.09)	0.34
Ever smoker (*n* = 318)	1 (ref)	0.78 (0.59, 1.03)		0.74 (0.57, 0.96)	
Kidney cancer					
Never smoker (*n* = 94)	1 (ref)	0.59 (0.34, 0.99)	0.04	0.77 (0.49, 1.21)	0.65
Ever smoker (*n* = 118)	1 (ref)	1.04 (0.66, 1.62)		0.78 (0.53, 1.15)	

All models used age (month) as time scale with stratification by calendar time (year) and adjusted for race (white or non-white), height (continuous), family history of cancer (yes or no), physical exam in past two years (yes or no), history of colonoscopy or sigmoidoscopy (yes or no), smoking in pack years (never smoker, 1–4.9, 5–19.9, 20–39.9 or ≥40), regular aspirin use (yes or no), multivitamin use (yes or no), alcohol consumption (0, 0.1–4.9, 5.0–14.9, 15.0–29.9 or ≥30 g/d), red and processed meat intake (quintiles), Alternate Healthy Eating Index (quintiles), prostate-specific antigen test in past two years (yes or no), and total physical activity except for resistance training (quintiles). For kidney cancer, high blood pressure (yes or no) was further adjusted.

^a^P-interaction was calculated by including a cross-product term of resistance training (binary) and each stratification variable.

^b^P-interaction was calculated by including a cross-product term of resistance training (continuous) and each stratification variable.

### Joint association of resistance training and total physical activity with cancer risk

We also evaluated the joint association of resistance training and total physical activity with risk of bladder, kidney and colon cancers (Table 4). Compared to participants with low physical activity and none resistance training, high physical activity and any resistance training was associated with a lower risk of kidney cancer (HR 0.65; 95% CI 0.43, 0.97). HR for kidney cancer comparing participants with high physical activity and none resistance training vs. low physical activity and none resistance training was 0.87 (95% CI 0.62, 1.21). There was no evidence of joint association of resistance training and total physical activity with bladder and colon cancers.

**Table 4:** Joint association of resistance training and total physical activity with risk of site-specific cancer in the Health Professionals Follow-up Study (1992–2014).

HR (95% CI)	Total physical activity (MET-h/week)^a^	
	Low	High
Colon cancer (*n* = 646)		
Event	216	280
None resistance training	1 (ref)	0.88 (0.73, 1.06)
Event	30	174
Any resistance training	0.84 (0.57, 1.25)	0.95 (0.77, 1.19)
Bladder cancer (*n* = 435)		
Event	130	215
None resistance training	1 (ref)	1.19 (0.95, 1.50)
Event	20	140
Any resistance training	0.75 (0.46, 1.21)	1.03 (0.80, 1.35)
Kidney cancer (*n* = 192)		
Event	68	79
None resistance training	1 (ref)	0.87 (0.62, 1.21)
Event	15	50
Any resistance training	1.02 (0.57, 1.83)	0.65 (0.43, 0.97)

All models used age (month) as time scale with stratification by calendar time (year) and adjusted for race (white or non-white), height (continuous), family history of cancer (yes or no), physical exam in past two years (yes or no), history of colonoscopy or sigmoidoscopy (yes or no), smoking in pack years (never smoker, 1–4.9, 5–19.9, 20–39.9 or ≥40), regular aspirin use (yes or no), multivitamin use (yes or no), alcohol consumption (0, 0.1–4.9, 5.0–14.9, 15.0–29.9 or ≥30 g/d), red and processed meat intake (quintiles), Alternate Healthy Eating Index (quintiles), and prostate-specific antigen test in past two years (yes or no). For kidney cancer, high blood pressure (yes or no) was further adjusted.

P-interaction:  0.26 for colon cancer; 0.55 for bladder cancer; 0.40 for kidney cancer.

^a^Low physical activity was defined as the lowest tertile (<16 MET-h/week) and high physical activity was defined as top-two tertiles (≥16 MET-h/week).

### Sensitivity analysis

We performed a sensitivity analysis using any vs. none resistance training categories (Table S1). Resistance training was not associated with total cancer risk (multivariable model 2: HR 0.98; 95% CI 0.91, 1.04). We also did not find an association between any vs. none resistance training for bladder cancer (HR 0.85; 95% CI 0.69, 1.05), kidney cancer (HR 0.80; 95% CI 0.57, 1.10) and colon cancer (HR 1.03; 95% CI 0.86, 1.23).

## Discussion

In this large prospective cohort study, resistance training was not associated with total cancer risk. However, we observed an association between resistance training and lower risk of bladder and kidney cancer after adjusting for potential confounders and total physical activity (excluding resistance training). Eight types of cancer presented null findings. Combined resistance training and high total physical activity was associated with lower risk of kidney cancer compared to none resistance training and low total physical activity.

The potential protective effect of physical activity on cancer has received great attention in the past few years. In 2018, The World Cancer Research Fund (WCRF) and an umbrella review of the literature acknowledged that MVPA reduces the risk of breast, colon and endometrial cancers.^1,2^ Importantly, the largest study conducted on this topic pooled data from 12 cohort studies, including over 187 thousand cancer cases, and found association between leisure-time physical activity and lower risk of 13 types of cancer (colon, breast, endometrial, oesophageal adenocarcinoma, liver, kidney, gastric cardia, myeloid leukaemia, myeloma, head and neck, rectal and bladder).^3^ Considering these findings, the advisory committees of the American College of Sports Medicine and the U.S. Department of Health and Human Services concluded that strong evidence supports a protective effect of physical activity on the cancers of the colon, breast, endometrium, kidney, bladder, oesophagus and stomach.^4^ Details on the optimum type of physical activity to reduce cancer risk have been understudied and thus considered a research priority by these organisations.^4,6^

Our study adds knowledge on a particular type of physical activity, resistance training, in relation to total and site-specific cancer risk. Overall, resistance training does not seem to have a protective effect on all types of cancer previously linked with MVPA. Our analyses indicated, however, that resistance training may have a protective effect on the cancers of the bladder and kidney. To our knowledge, only one prospective cohort study performed similar analysis on muscle-strengthening activities and cancer incidence. Mazzilli and colleagues examined the association between weight training and risk of 10 common type of cancer in a large cohort of 313,363 middle-aged and older adults, the National Institutes of Health (NIH)-American Association of Retired Persons (AARP) Diet and Health Study.^7^ Weight lifting was associated with lower risk of colon cancer, and a trend towards lower risk of kidney cancer. Our findings are in agreement with the NIH-AARP Diet and Health study, except for colon cancer. In our study, we found an unexpected positive association between resistance training and colon cancer after adding total physical activity, total caloric intake and BMI into the model. Subgroup analysis by BMI and smoking showed that resistance training was associated with increased risk of colon cancer only among ever smokers and overweight/obese participants, although interactions were not statistically significant. Null findings were observed in the joint association of resistance training and total physical activity with colon cancer.

There are few candidate biological mechanisms linking resistance training and kidney and bladder cancer. It has been speculated that resistance training may reduce the risk of cancer by maintaining glucose homeostasis and lowering insulin and insulin resistance, which can stimulate cell proliferation and inhibit apoptosis.^15^ However, if this is the main biological mechanism, we would expect to observe inverse associations of resistance training with other insulin-related cancers (e.g. colon and pancreas).^16^ In addition, a cross-sectional study including 7219 men in the HPFS found that combined resistance training and aerobic activities are associated with favourable biomarkers of inflammation and insulin-response compared to those engaging in aerobic only.^17^ However, systemic changes of lipids, inflammatory markers, sex hormones, metabolic hormones and growth factors (GH, IGF-1) have not been observed in clinical intervention studies including cancer patients.^18^ Resistance training may also lower blood pressure, thus supporting the inverse association with kidney cancer.^19^ Finally, resistance training has been linked with activation of molecular pathways linked to cell growth and metabolism (mTOR) dysregulated during cancer progression.^20^

Our study has several limitations. First, the magnitude of the associations is likely underestimated due to measurement error in self-reported resistance training.^8^ However, we used repeated-measures, and calculated cumulative average of resistance training over time, which allows to capture long-term exposure and reduces measurement error. Second, low variability of resistance training within participants in our cohort may have contributed to null findings. On the other hand, cumulative average of resistance training has been associated with diabetes and coronary heart disease in the HPFS.^15,20^ These findings support that measurement of resistance training in our cohort is sufficient to predict some health outcomes, but any association with cancer, if causal, is likely weaker than for other endpoints. Third, we did not collect information on the participants’ resistance training regimes (weekly frequency and intensity). Fourth, 10,890 participants did not report information on resistance training and were excluded from our analysis, which might have introduced selection bias if lack of information is systematically associated with both exposure and cancer risk. However, we compared missing data group vs. those with completed resistance training questionnaire and they presented similar distribution of basic characteristics (e.g. age, BMI, smoking status, calorie intake, diet quality), providing evidence against selection bias (data not shown). Fifth, although we applied a 2-year time lag and comprehensively adjusted for potential confounders, reverse causation and residual confounding may still be present. Finally, we may have limited power to identify some associations between resistance training and less common cancers.

In conclusion, resistance training was not associated with total cancer risk, but it was inversely associated with bladder and kidney cancers. Combined resistance training and aerobic activities suggested additional risk reductions for kidney cancer. Future large prospective cohort studies and pooled data analysis are warranted to confirm our findings.

## Supplementary information

Table S1

## Data Availability

The data that support the findings of this study are available from the corresponding author upon reasonable request.
