# Supplementary material for: Resistance training and total and site-specific cancer risk: a prospective cohort study of 33,787 US men
Source: Br J Cancer. 2020 Jun 4;123(4):666–72. doi: 10.1038/s41416-020-0921-8 (PMC7434758; doi:10.1038/s41416-020-0921-8)
Supplement: Supplementary file 1 — Table S1 [file 41416_2020_921_MOESM1_ESM.docx]

**SUPPLEMENTARY MATERIALS**

| **Table S1. Resistance training (none vs. any) and risk of total and site-specific cancer in the Health Professionals Follow-up Study (1992-2014)** | | | |  |
| --- | --- | --- | --- | --- |
|  | **Resistance training** | | **Per 1-hour/wk increase** | |
| HR (95% CI) | **None** | **Any** |  |  |
| **Total cancer** (n=5158)^a^ |  |  |  | |
| Event | 3 530 | 1 628 |  | |
| Person years | 336 057 | 185 164 |  | |
| Multivariable 1 | 1 (ref) | 0.97 (0.91, 1.03) | 1.00 (0.96, 1.04) | |
| Multivariable 2 | 1 (ref) | 0.98 (0.91, 1.04) | 1.01 (0.97, 1.05) | |
| Multivariable 3 | 1 (ref) | 0.98 (0.92, 1.05) | 1.01 (0.97, 1.05) | |
| **Cancer sites** |  |  |  | |
| Colon cancer (n=700) |  |  |  | |
| Event | 496 | 204 |  | |
| Multivariable 1 | 1 (ref) | 1.01 (0.85, 1.21) | 1.10 (1.00, 1.20) | |
| Multivariable 2 | 1 (ref) | 1.03 (0.86, 1.23) | 1.11 (1.02, 1.22) | |
| Multivariable 3 | 1 (ref) | 1.04 (0.87, 1.25) | 1.12 (1.02, 1.22) | |
| Advanced prostate cancer (n=657) |  |  |  | |
| Event | 487 | 170 |  | |
| Multivariable 1 | 1 (ref) | 0.93 (0.77, 1.13) | 0.95 (0.84, 1.07) | |
| Multivariable 2 | 1 (ref) | 0.93 (0.77, 1.13) | 0.95 (0.84, 1.07) | |
| Multivariable 3 | 1 (ref) | 0.93 (0.77, 1.13) | 0.95 (0.84, 1.07) | |
| Lung cancer (n=595) |  |  |  | |
| Event | 447 | 148 |  | |
| Multivariable 1 | 1 (ref) | 0.86 (0.70, 1.05) | 0.91 (0.78, 1.07) | |
| Multivariable 2 | 1 (ref) | 0.88 (0.71, 1.08) | 0.93 (0.79, 1.09) | |
| Multivariable 3 | 1 (ref) | 0.87 (0.71, 1.07) | 0.93 (0.79, 1.09) | |
| Bladder cancer (n=505) |  |  |  | |
| Event | 345 | 160 |  | |
| Multivariable 1 | 1 (ref) | 0.88 (0.72, 1.08) | 0.81 (0.68, 0.97) | |
| Multivariable 2 | 1 (ref) | 0.85 (0.69, 1.05) | 0.80 (0.66, 0.96) | |
| Multivariable 3 | 1 (ref) | 0.85 (0.69, 1.05) | 0.80 (0.66, 0.96) | |
| Lymphoma (n=484) |  |  |  | |
| Event | 316 | 168 |  | |
| Multivariable 1 | 1 (ref) | 1.02 (0.83, 1.26) | 1.06 (0.94, 1.18) | |
| Multivariable 2 | 1 (ref) | 1.04 (0.84, 1.29) | 1.06 (0.95, 1.19) | |
| Multivariable 3 | 1 (ref) | 1.04 (0.84, 1.29) | 1.07 (0.95, 1.19) | |
| Pancreatic cancer (n=233) |  |  |  | |
| Event | 153 | 80 |  | |
| Multivariable 1 | 1 (ref) | 1.07 (0.79, 1.43) | 0.98 (0.80, 1.19) | |
| Multivariable 2 | 1 (ref) | 1.13 (0.83, 1.53) | 1.01 (0.83, 1.23) | |
| Multivariable 3 | 1 (ref) | 1.15 (0.85, 1.56) | 1.01 (0.84, 1.23) | |
| Kidney cancer (n=212) |  |  |  | |
| Event | 147 | 65 |  | |
| Multivariable 1 | 1 (ref) | 0.76 (0.55, 1.04) | 0.74 (0.56, 0.99) | |
| Multivariable 2 | 1 (ref) | 0.80 (0.57, 1.10) | 0.77 (0.58, 1.03) | |
| Multivariable 3 | 1 (ref) | 0.80 (0.58, 1.11) | 0.78 (0.58, 1.04) | |
| Leukemia (n=188) |  |  |  | |
| Event | 129 | 59 |  | |
| Multivariable 1 | 1 (ref) | 0.82 (0.58, 1.14) | 1.05 (0.87, 1.27) | |
| Multivariable 2 | 1 (ref) | 0.85 (0.60, 1.20) | 1.09 (0.90, 1.32) | |
| Multivariable 3 | 1 (ref) | 0.86 (0.61, 1.21) | 1.09 (0.90, 1.32) | |
| Multiple myeloma (n=112) |  |  |  | |
| Event | 75 | 37 |  | |
| Multivariable 1 | 1 (ref) | 0.95 (0.62, 1.48) | 0.84 (0.58, 1.21) | |
| Multivariable 2 | 1 (ref) | 0.96 (0.62, 1.50) | 0.85 (0.58, 1.23) | |
| Multivariable 3 | 1 (ref) | 0.97 (0.62, 1.52) | 0.85 (0.59, 1.24) | |
| Esophageal (n=103) |  |  |  | |
| Event | 69 | 34 |  | |
| Multivariable 1 | 1 (ref) | 1.13 (0.71, 1.79) | 0.91 (0.64, 1.29) | |
| Multivariable 2 | 1 (ref) | 1.13 (0.71, 1.81) | 0.92 (0.65, 1.30) | |
| Multivariable 3 | 1 (ref) | 1.12 (0.70, 1.79) | 0.91 (0.64, 1.30) | |
| Age-adjusted models: Cox regression models using age (month) as time scale with stratification by calendar time (year).  Multivariable 1 models: Additionally adjusted for race (white or non-white), height (continuous), family history of cancer (yes or no), physical exam in past two years (yes or no), history of colonoscopy or sigmoidoscopy (yes or no), smoking in pack years (never smoker, 1-4.9, 5-19.9, 20-39.9, or ≥40), regular aspirin use (yes or no), multivitamin use (yes or no), alcohol consumption (0, 0.1-4.9, 5.0-14.9, 15.0-29.9, or ≥30 g/d), red and processed meat intake (quintiles), Alternate Healthy Eating Index (quintiles) and prostate specific antigen test in past two years (yes or no).  Multivariable 2 models: Additionally adjusted for total physical activity except for resistance training (quintiles).  Multivariable 3 models: Additionally adjusted for total energy intake (quintiles) and body mass index (quintiles).  ^a^ Included only aggressive prostate cancer as total cancer. | | | |  |
